# How to check and record a patient's body temperature

**Published:** 2013

**Authors:** Dianne Pickering

**Affiliations:** Nurse Advisor (retired), Community Eye Health Journal dianne_logan@hotmail.com

**Figure:**
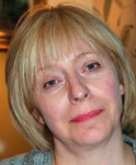
Dianne Pickering

All patients must be assessed for fitness before they can undergo surgery. As part of this assessment it is important to check and record the patient's temperature.

There are two reasons for this:

- It provides an initial recording (a ‘baseline’). If the temperature rises above this level after surgery, we are alerted to the fact that the patient may have an infection.
- It allows us to confirm that it is safe to operate on the patient. A high temperature may suggest an infection, which will have to be treated before the patient can undergo surgery.

## Clinical glass thermometers

This article will cover checking the temperature using a clinical glass thermometer which contains mercury. This is the most accurate and most commonly used thermometer. Digital thermometers are available but they require batteries which may not be readily available.

**WARNING:** Mercury is toxic. If a thermometer breaks, put on gloves and use a tissue or a piece of cloth to dispose of the mercury carefully, e.g., into a sharps bin. Do not allow it to be handled or get into water supplies.

## What is a normal body temperature?

The normal range for human body temperature is between 36°C and 38°C. However, it is usual to consider a reading above 37.2°C as suspicious. Repeat checks should be done.

A patient's temperature may be altered due to hormonal changes, exposure to heat or cold, exercise, and infection.

## You will need

- A clinical glass thermometer
- Disinfectant or an alcohol wipe
- A watch or clock
- Tissue or dry swab
- Record chart /patient's notes
- Pen

## Preparation

1. Wash and dry your hands – this will help to prevent cross-infection.
2. Explain to the patient what you are going to do. This will help the patient to understand and will make it easier for them to cooperate.
3. Disinfect the thermometer by wiping with an alcohol wipe, or use a swab moistened with the disinfectant. Dry with a tissue or swab.

## Method

We recommend taking the temperature in the axilla (armpit) as this is the easiest and safest place.

1. Ask the patient to loosen any tight clothing or remove long-sleeved garments so it is possible to access the axilla.
2. Hold the thermometer at the upper end. Shake it to ensure all the mercury is at the bottom. Clinical glass thermometers have a constriction in the tube so that once the mercury is above the constriction it cannot go down again until shaken. If you do not shake the thermometer it will result in an inaccurate reading.
3. Place the thermometer in the axilla (armpit). Place the forearm across the chest and ensure the upper arm is resting against the patient's side.
4. Leave the thermometer in place for 5 minutes. This will ensure that the reading will be accurate.
5. Remove the thermometer, read, and immediately record the temperature on the record chart or in the patient's notes.
  **NOTE:** The thermometer will cool down when exposed to the air, so read the temperature immediately to avoid a low and false recording.
6. Tell the patient the temperature and whether any further investigations are needed.
7. Disinfect the thermometer and wash and dry your hands again.
8. Report a raised temperature to the clinical person in charge.

**Figure:**
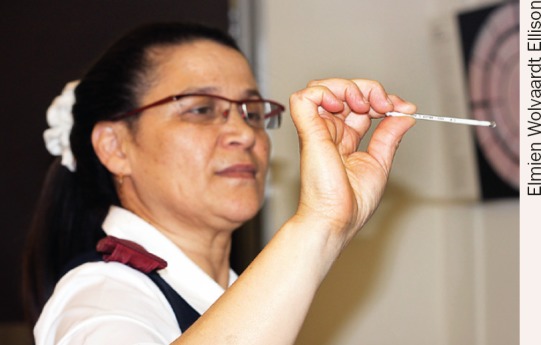
Read the temperature immediately after removing it from the patient's axilla (armpit).

**Figure:**
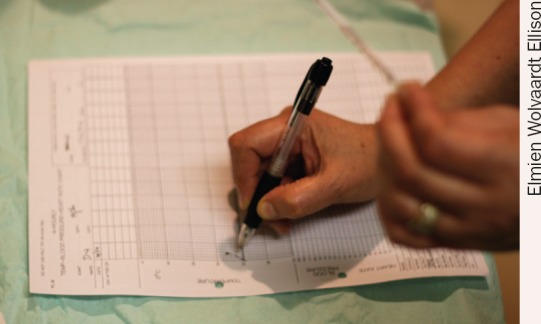
Record the temperature on the record chart or in the patient's notes.

